# Complete response of refractory tumid lupus to upadacitinib

**DOI:** 10.1016/j.jdcr.2026.08.047

**Published:** 2026-09-04

**Authors:** Ella Engels, Christopher T. Richardson

**Affiliations:** a University of Rochester School of Medicine and Dentistry, Rochester, New York; b Department of Dermatology, University of Rochester Medicine, Rochester, New York; c Division of Allergy, University of Rochester Medicine, Immunology and Rheumatology, Rochester, New York

**Keywords:** cutaneous lupus erythematosus, Janus kinase inhibitor, lupus erythematosus tumidus, upadacitinib

## Introduction

Lupus erythematosus tumidus (LET) is a photosensitive subtype of cutaneous lupus erythematosus (CLE) characterized by urticarial-like, erythematous, nonscarring annular plaques^1^ with minimal epidermal surface changes (eg, scale or follicular plugging).^2^ LET is most commonly found on the face, upper neck, trunk and upper extremity extensor surfaces.^2^ The clinical course of LET often includes cyclic relapse and remission without scarring or dyspigmentation.^3^

Treatment of LET typically includes photoprotection, topical corticosteroids, and intralesional corticosteroids as well as antimalarial therapy (eg, hydroxychloroquine).^2^ Alternative therapies include immunosuppressants such as mycophenolate or methotrexate.^4^ Currently, there are no biologic therapies approved specifically for CLE, including the subtype of LET. In this report, we discuss a case of LET refractory to topical and intralesional steroids, antimalarials, and mycophenolate that showed a complete response to the Janus kinase inhibitor (JAKi) upadacitinib.

## Case report

A 49-year-old man presented with a 1-year history of an asymptomatic red rash on his shoulders that flared intermittently throughout the year. Physical exam showed multiple dull pink firm annular and circular plaques without overlying scale on bilateral shoulders (Fig 1). Social history is significant for smoking up to 5 cigars daily.

**Fig 1:**
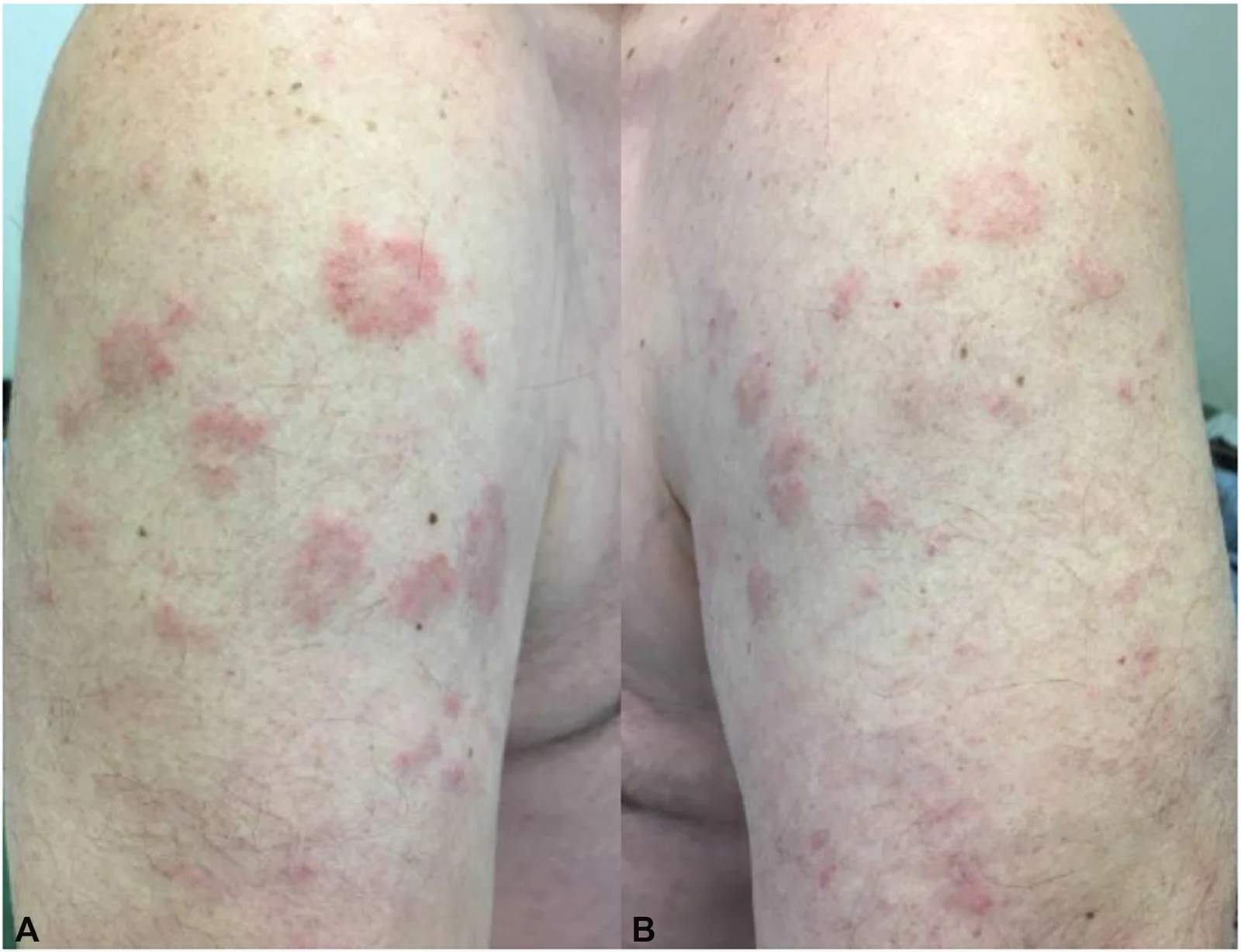
Right **(A)** and left **(B)** shoulders with multiple pink annular plaques without overlying scale.

A right upper shoulder biopsy at the time of onset showed superficial and deep perivascular lymphocytic infiltrate with an increase in interstitial mucin, most consistent with LET. Repeat skin biopsy at 1 year revealed epidermal atrophy, lymphocytic infiltrate and increased dermal mucin, again consistent with cutaneous lupus. Direct immunofluorescence was negative. Laboratory testing revealed a positive antinuclear antibody (1:80) and anticardiolipin immunoglobulin M antibody (with no history of thromboses). All other serologies were otherwise negative.

The patient had been treated previously with hydroxychloroquine, which was discontinued due to gastrointestinal side effects, and oral steroids. He initially achieved clinical remission with clobetasol cream 0.05% twice daily as needed and quinacrine 100 mg daily. After 8 months of quinacrine therapy, discontinuation for 1 month resulted in disease recurrence involving the shoulders as well as expansion to the upper back (Fig 2). This flare resolved with resumption of quinacrine along with triamcinolone 0.1% cream for flares. Following an additional 8 months of remission, quinacrine was discontinued due to a national shortage and inability to obtain the medication, and the patient was transitioned to chloroquine 250 mg daily.

**Fig 2:**
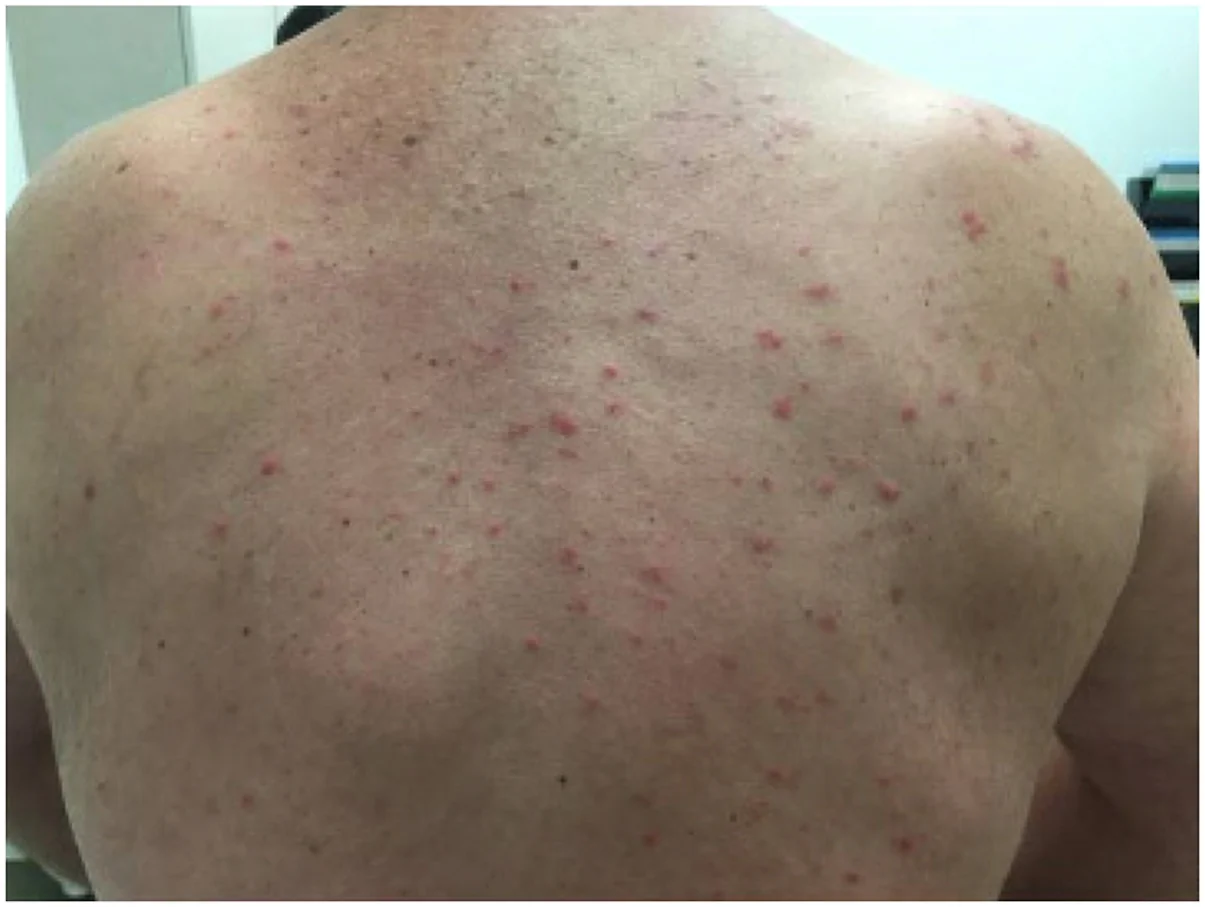
Back with several erythematous non-scaly papules after discontinuation of quinacrine 100 mg daily.

Over the subsequent 18 months, he experienced progressively more frequent and extensive flares involving expansion from the shoulders and upper back to the chest and anterior neck in the setting of multiple failed treatments (Table I). Fluorouracil cream was trialed once for an LET flare by another provider but worsened the eruption. Mycophenolate mofetil up to 1500 mg twice daily failed to adequately control disease activity. The patient was not a candidate for methotrexate as he engages in daily alcohol use. Intralesional triamcinolone (0.7 mL of 10 mg/mL) was also trialed with a single injection into a lesion on the left shoulder, which was ineffective.

**Table I:** Failed treatments

Medication	Maximum dose	Duration	Efficacy/side effects
Antimalarials			
Hydroxychloroquine	400 mg d	Unknown	GI side effects
Quinacrine	100 mg d	17 mo	Effective, but unavailable
Chloroquine	250 mg d	46 mo	Breakthrough flares
Topicals			
Clobetasol	0.05% twice d prn	2 mo	Breakthrough flares
Triamcinolone	0.1% twice d prn	29 mo	Ineffective
Fluorouracil	Unknown – prescribed by outside provider	Once	Worsened
Intralesional steroids			
Triamcinolone	0.7 mL of 10 mg/mL	Once	Breakthrough flares
Immunosuppressants			
Mycophenolate mofetil	1000-3000 mg d	22 mo	Breakthrough flares
Biologics			
Ixekizumab	80 mg mo	5 mo	Breakthrough flares
Bimekizumab	160 mg every 28 d	8 mo	Breakthrough flares

The patient was subsequently diagnosed with ankylosing spondylitis and treated with ixekizumab for 5 months (with no effect on the LET) before transitioning to upadacitinib 15 mg daily. During this period, chloroquine was discontinued. While receiving upadacitinib, the patient remained free of LET flares for over 2 years without the need for other systemic or topical medications. However, the patient achieved only partial relief of his back pain and was switched from upadacitinib to bimekizumab for better management of ankylosing spondylitis. While on bimekizumab, LET flares recurred within 8 months and he did not feel his joint symptoms improved. Given the loss of LET control and lack of improvement in joint pain, the patient was switched back to upadacitinib 15–30 mg daily and has remained free of LET lesions ever since (Fig 3). Complete blood count, comprehensive metabolic panel, and Sjogren’s syndrome antigen A and B autoantibodies have been unremarkable while on therapy.

**Fig 3:**
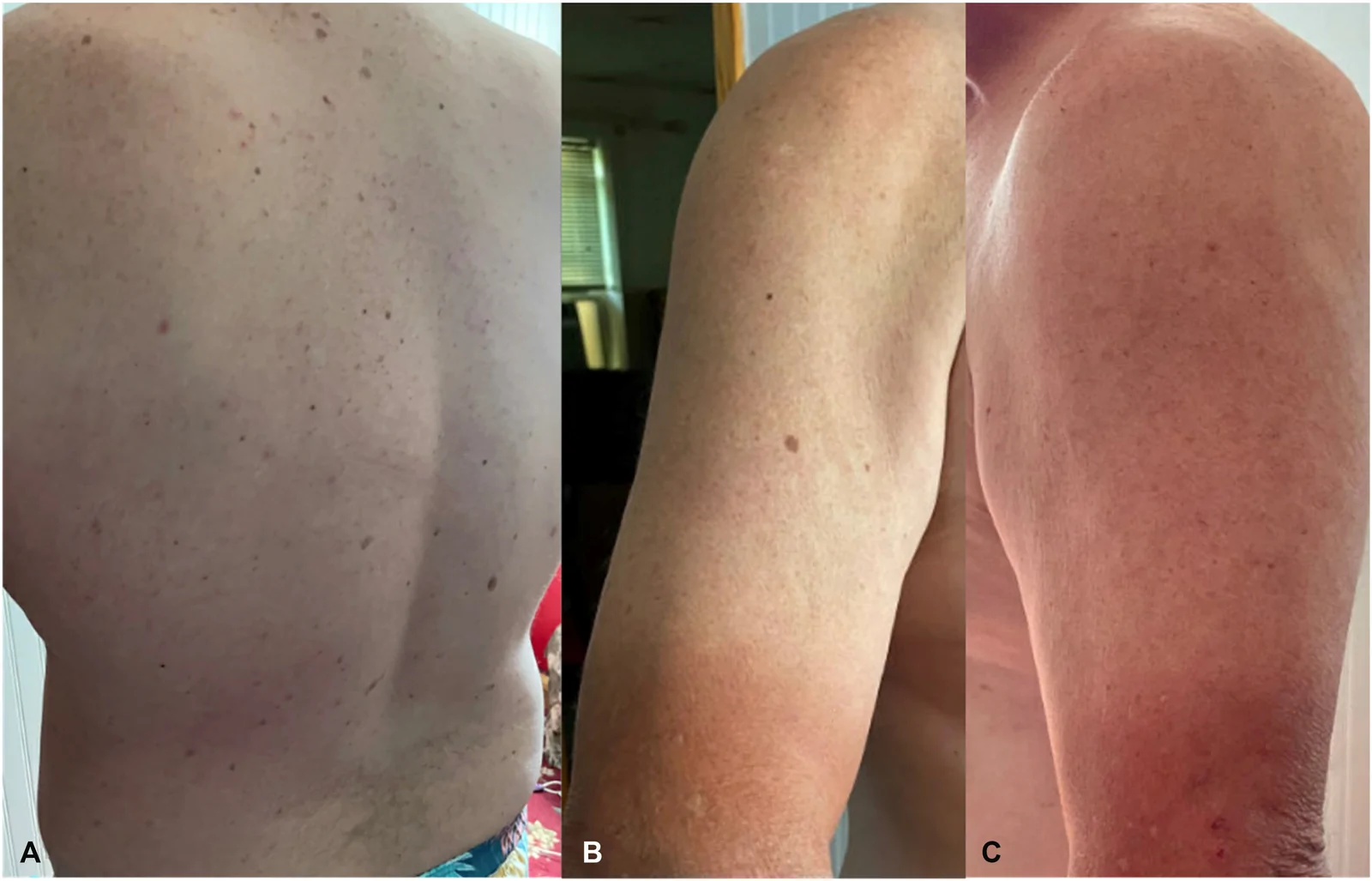
Back **(A)**, right shoulder **(B)**, and left shoulder **(C)** without LET flares since resuming upadacitinib.

## Discussion

This patient represents the first published case, to the best of our knowledge, of LET refractory to multiple systemic treatments and successfully treated with upadacitinib. JAKi are currently United States Food and Drug Administration approved for rheumatic disorders including rheumatoid arthritis, psoriatic arthritis, and ankylosing spondylitis as well as dermatologic conditions including atopic dermatitis. Their use in treating complex inflammatory dermatologic disorders is becoming more evident as dysregulation of JAK-mediated signaling pathways contributes to many immune-related dermatologic conditions.^5^ There are reports of off-label JAKi use for other CLE subtypes refractory to first line treatments, including baricitinib for familial chilblain lupus and tofacitinib for cutaneous lupus.^5^^,^^6^ However, there are no reports of JAKi used for LET.

The pathogenesis of CLE is driven by interferons, which signal through the JAK-mediated signaling pathway.^7^ A preclinical study showed that JAK1 inhibition significantly reduces expression of proinflammatory mediators in CLE in vitro models and improved skin lesions in lupus-prone mice.^7^ Upadacitinib is a selective JAK1 inhibitor, which suggests it has favorable selectivity for treating CLE and its associated subtypes, including LET. Upadacitinib is currently in the phase III trial for systemic lupus erythematosus and has been shown to improve the Cutaneous Lupus Erythematosus Disease Area and Sensitivity Index-50 response and cutaneous symptoms.^8^ Another phase II study consisting of 341 patients showed that upadacitinib 30 mg alone or in combination with elsubrutinib (Bruton tyrosine kinase inhibitor) resulted in significant improvements in SLE disease through flare reduction.^9^

This case suggests that oral JAK1 inhibition with upadacitinib may provide an effective alternative for refractory LET when antimalarials, immunosuppressants, and steroids have failed. That said, as a single case report, this observation cannot establish generalizability. The optimal dose, duration of therapy, and long-term outcomes of upadacitinib in treating LET are unknown. Future studies are needed to validate efficacy of upadacitinib in treating CLE subtypes, including LET.

## Conflicts of interest

Richardson has served as an investigator for AstraZeneca, Incyte, LEO Pharma, Priovant and Ribon Therapeutics and as a consultant for Biogen, Immunovant, Merck, Pfizer and Priovant. Engels has no conflicts of interest or disclosures.
